# Nutritional, Physicochemical, Microstructural, Rheological, and Organoleptical Characteristics of Ice Cream Incorporating *Adansonia digitata* Pulp Flour

**DOI:** 10.3390/foods12030533

**Published:** 2023-01-25

**Authors:** Sally S. Sakr, Sahar H. S. Mohamed, Asmahan A. Ali, Waheeba E. Ahmed, Reham M. Algheshairy, Mona S. Almujaydil, Ahmed A. Al-Hassan, Hassan Barakat, Mohamed F. Y. Hassan

**Affiliations:** 1 Department of Food Science and Human Nutrition, College of Agriculture and Veterinary Medicine, Qassim University, Buraydah 51452, Saudi Arabia; 2 Dairy Science Department, Faculty of Agriculture, Cairo University, Giza 12613, Egypt; 3 Dairy Department, Food Industries and Nutrition Research Institute, National Research Centre, 33rd Buhouth Street, Dokkii, Giza 12622, Egypt; 4 Department of Biotechnology and Food Safety, National Food Research Centre, Khartoum P.O. Box 213, Sudan; 5 Department of Food Science and Technology, Alzaiem Alazhari University, Khartoum 1432, Sudan; 6 Food Technology Department, Faculty of Agriculture, Benha University, Moshtohor 13736, Egypt; 7 Department of Dairy Science, Faculty of Agriculture, Sohag University, Sohag 82755, Egypt

**Keywords:** ice cream, *Adansonia digitata*, nutrition, antioxidants, rheology, microstructure

## Abstract

Ice cream’s appeal is unrivaled. Nonmilk and milk ingredients in ice cream formulas affect their nutritional value, structure, and organoleptical qualities. Seeking novel dietary ingredients instead of artificial flavoring compounds is vital for improving ice cream taste preference, adding antioxidants, and increasing nutritional value. The current study examines the feasibility of manufacturing a new flavored ice cream with excellent dietary value using *Adansonia digitata* L. (Baobab) fruit pulp (ADFP). The prepared ice cream’s physicochemical and microbiological quality, and rheological, microstructural, and organoleptic properties were investigated. Using ADFP instead of skim milk powder with a partial or complete replacement, five ice cream samples were produced and marked as IB-0, IB-25, IB-50, IB-75, and IB-100. Chemical characteristics were not noticeably impacted except protein and ash, which considerably decreased with increasing ADFP levels. Increasing ADFP in the samples increased titratable acidity and reduced pH. All ice cream samples were microbiologically acceptable with no pathogenic bacteria. By increasing ADFP in the samples, the daily values (%DV) of sodium, potassium, and magnesium were not considerably affected. Calcium reduced from 14.91% in IB-0 to 7.75% in IB-100. All microelements found in the study rose considerably as ADFP increased. Increasing ADFP levels significantly boosted antioxidant levels. The IB-100 sample had the highest total phenolic content (149.29 mg GAE 100 g^−1^), antioxidant activity (98.12 µmol of TE 100 g^−1^), total flavonoids (5.96 mg QE 100 g^−1^), and total flavanols (4.01 mg QE 100 g^−1^). The inclusion of ADFP had a beneficial effect on the color of the samples. It did not negatively affect the ice cream’s organoleptic acceptability as determined by organoleptic, rheological, and microstructural examinations. Interestingly, when skim milk powder was replaced with 50% and 75%, the hardness, viscosity, and aqueous phase separation were significantly improved. In conclusion, ADPF improves the nutritional value of ice cream and can be used as a natural coloring ingredient without affecting microstructural and rheological properties.

## 1. Introduction

Supply for progress and healthful products is periodically bolstered to meet the populace’s needs. Thus, food with additional bioactive components is developed and labeled by food manufacturers to give fundamental nutrients and health advantages in terms of “functional foods” [1]. Extra nutritional health benefits related to dairy consumption have been documented [2]. Energy, calcium, proteins, and other micro- and macronutrients are vital substances that dairy products contribute to our diet [3]. Ice cream is globally one of the most popular dairy products [4]. Nonetheless, commercial ice cream is typically deficient in natural antioxidants such as vitamin C, natural colors, and polyphenols. Fruits and vegetables are rich in natural antioxidants, natural dyes, and vitamins, low in fat, and free from synthetic additions, offering a practical way to increase the nutritional content of ice cream [5,6]; even manufacturing ice cream incorporating probiotics [7] was considered.

The production procedures of blending, pasteurizing, homogenizing, cooling, aging, flavoring and coloring, whipping, packing, and hardening are all crucial to the final ice cream product. The base ingredients are milk fat and milk solids not-fat (SNF), sugar, water, stabilizers, emulsifiers, and 0.3% liquid flavors and colors [8]. Protein adsorbed to the surface of fat globules must be partially desorbed during the aging stage. Melting point and overrun are crucial factors to consider when assessing an ice cream product and its manufacturing process [9]. Fruits and vegetables contain dietary fibers and polysaccharides that have significant melting resist potential with unremarkable effects on viscosity, overrun, and texture [10] because of their water-binding capacity [11].

The stately tree *Adansonia digitata* L. (*A. digitata*), known as baobab, is a member of the Malvaceae family, and is highly valued for its medicinal and nutritional benefits [12]. It can withstand hot weather and dry spells, and is cultivated for its sour fruit and leaves. Each part of the *A. digitata* plant—seeds, leaves, roots, blossoms, fruit pulp, and bark—can be eaten, and used in cooking and baking [12,13,14]. The fruit’s dry, acidic pulp and large seeds are used in cooking and beverage making [15]. The fruit pulp has a vitamin C value nearly 10 times that of oranges, along with carbohydrates and a high concentration of pectin [14]. There is much interest in the antioxidant and anti-inflammatory effects of various sections of this tree; thus, it is frequently used in alternative medicine [16]. The pulp and seeds were analyzed for their approximate composition and mineral concentration [17]. Significant K, Ca, Mg, Na, P, Fe, Zn, and fiber levels were found. According to Hyacinthe et al. [18], *A. digitata* is rich in beneficial nutrients such as vitamins B1 and B2, and minerals. Seeds are high in nutrients such as protein and fat, and can be used as a food source. The chemical composition of 20 kinds of *A. digitata* indicated 1250–12.91, 4.75–5.18, 2.24–2.42, and 52.17–56.62% for moisture, ash, protein, and total dietary fibers, respectively [19]. The sulfur-containing amino acids were weakest in quantity, while glutamic and aspartic acid levels were high [14,20]. According to the fatty acid profile, oleic and linoleic acids were the most prevalent unsaturated fatty acids, whereas palmitic acid was the most pervasive saturated acid [14]. *A. digitata* fruit pulp (ADFP) primarily consisted of hydroxycinnamic acid glycosides, iridoid glycosides, and phenylethanoid glycosides [21]. An HPLC investigation showed that *A. digitata* contained significant amounts of flavonoids and phenolic acids such as catechin, quercitrin, quercetin, epicatechin, luteolin, rutin, kaempferol, gallic, caffeic, chlorogenic, and ellagic acids [22]. According to Sokeng et al. [23], profiling the primary and secondary metabolites of baobab fruits and leaves fills in the gaps in our understanding of the plant’s chemical makeup, lends credence to the growing body of evidence attesting to baobab’s nutritional and biological properties, and offers guidance on how these ingredients might be put to use in future applications such as food, medicine, and personal care. Scientists have isolated 46 different phytochemicals from fruit pulp, most of which are proanthocyanidins, phenolic acids, flavonols, and saponins [24].

Ismail et al. [25] showed that baobab fruit shells (BFSs) have much potential as an abundant supply of phenolic chemicals, with potential uses in the food and pharmaceutical industries. Carbohydrate and lipid metabolisms were both boosted by ADFP and leaf extracts [22,26,27]. Weight loss, anti-inflammatory, hypolipidemic, hypoglycemic, renal, hepatic, and cardioprotective effects were among the benefits of its aqueous extract for people with metabolic syndrome [28,29] besides relieving pain [30]. In silico testing by Adeoye et al. [31] showed that the secondary metabolites apigenin and quercetin, both found in the ethylacetate-partitioned fraction of *A. digitata* stem bark, had antimalarial efficacy. There was encouraging evidence that eating baobab could help in curbing cravings and promote weight maintenance [32]. Hanafy et al. [33] discovered that *A. digitata* extracts significantly mitigated lipid peroxidation, which acted as a protective mechanism against hepatotoxicity. At 200 mg daily, ADFP had a cardioprotective effect against induced oxidative stress and led to mostly typical myocardial structure [34]. The flavonoids found in *A. digitata* have powerful antioxidant properties [35] that might not be able to restrict carotenoids’ bioavailability [36]. Procyanidins and flavonol glycosides, especially tiliroside, are abundant in *A. digitata* fruit pulp, rendering it a potential source of health-promoting compounds [37]. Baobab seed extract (BSE) was added to beef patties to increase their shelf life, and the consequent antioxidant and antibacterial activity increased lipid stability and maintained quality [38]. Incorporating baobab milk and fermented baobab/acha (African grain) flour into the meals of a low-income population is brilliant because the combined product is more nutritious than either component alone [39].

Interestingly, to the best of the authors’ knowledge, the literature review mainly highlighted that adding ADFP into ice cream has not been investigated so far. Therefore, the current study examines the feasibility of producing a new ice cream product by incorporating a substantial amount of ADFP. To commercially scale up ice cream, the nutritional, physicochemical, structural, rheological, microbiological, and organoleptic properties of prepared ice cream were investigated.

## 2. Materials and Methods

### 2.1. Materials

Raw cow milk (3.3% fat) from the herd of the faculty of agriculture and veterinary medicine, Qassim University, Al-Qassim, KSA was obtained. Fresh bulk milk was separated, and cream (55% fat) was collected at the processing unit of the Department of Food Science and Human Nutrition. Skim milk powder (95% solids not fat) was supplied by Arla Foods Company (Viby J, Denmark). Sugar, gelatin, and vanilla powders were purchased from the local market at Buraydah, Al-Qassim region, Saudi Arabia (SA). A Sudanese source of baobab fruit was obtained from the local market in Jeddah, SA. To extract the coated seeds and dry pulp, dried fruit was washed, and shells were cracked open before grinding using an electronic grinder (Severin, type Km 3871, Mecklenburg, Germany). The resultant ADFP was then sieved over a 60-mesh screen to obtain a fine, uniform powder that was immediately stored in dark glass jars at a temperature of 18 ± 1 °C until the formulation of different ice cream mixes.

### 2.2. Formulation of Different Ice Cream Mixes and Preparation of Ice Cream Samples

According to Arbuckle [9], five ice cream mixes were formulated using the ingredients listed in Table 1. Each mix’s liquid ingredients were first heated until the temperature had reached about 60 °C, and dried ingredients and solubilized gelatin were added. Then, the heat treatment (85 °C, 15 min) was completed with continuous stirring and cooled rapidly to prepare for the aging step. Cooled ice cream mixes were stored at 4 ± 1 °C for 24 h before the whipping process. The prepared five ice cream mixes were whipped at speed 6 and −7 °C for 5–6 min. Using an automated ice cream whipping machine (Finamac, Pro 16, Made By Renan Florencio Correia Silva, Santo André, Brazil) and the five related ice cream samples (IB-0, IB-25, IB-50, IB-75, and IB-100) containing different ADFP amounts, replaced skim milk powder was produced and kept at −18 °C until analysis. A portion of each ice cream sample was also freeze-dried (CHRIST, Alpha 1-2 L.D. plus, Germany) for 96 h at −48 °C under pressure of 0.032 mbar for transmission electron microscope (TEM) examination.

### 2.3. Methods of Analysis

#### 2.3.1. Chemical Composition

Standard AOAC (2005) procedures were used to determine the total solids, crude protein, fat, ash, and available carbohydrates [40].

#### 2.3.2. pH and Titratable Acidity (TA%)

The pH values of the different ice cream samples were detected using a pH meter (Jenway 3505, Staffordshire, UK). Titratable acidity (g lactic acid 100 mL^−1^) was determined following standard AOAC methods [40].

#### 2.3.3. Microbiological Quality of Different Ice Cream Formulas

The day after processing, the different ice cream samples and their mixes were evaluated for total bacterial count, coliform, and Staphylococcus aureus. Ice cream samples were left at room temperature for 10 min to facilitate the sampling step. Each ice cream sample (25 g) was homogenized with 225 mL peptone water; then, serial dilutions were performed in a ratio of 1:9 *v*/*v*. Samples thus prepared were processed for the following bacteriological determinations: (a) the total bacterial count was enumerated according to the conventional method (FDA, [41]) on plate count agar (Oxoid), incubated at 37 ± 1 °C for 48 h; (b) *Staphylococcus aureus* was enumerated according to ISO [42] on a Baird Parker agar base supplemented with egg yolk tellurite emulsion, incubated at 37 ± 1 °C for 48 h; (c) the coliform group was determined using the solid medium method onto plates of MacConkey agar (Merck, Darmstadt, Germany) according to the method reported by the FDA [41].

#### 2.3.4. Mineral Content

Mineral constituents (Ca, K, Na, Mg, P, Zn, and Fe) were determined using an atomic absorption flame emission spectrophotometer (Perkin-Elmer model AA-6200 from Shimadzu 7000, Tokyo, Japan). In addition, 1000 mg L^−1^ multielement certified standard solution (Merck, Germany) was used as a stock solution for instrument standardization as reported by AOAC [40].

#### 2.3.5. Total Phenolic Content, Antioxidant Activity, Total Flavonoids (TF), and Total Flavonols (TFL)

The prepared samples of ice cream were analyzed for TPC with the Folin–Ciocalteu reagent following a modified procedure developed by Bettaieb et al. [43]. In summary, freeze-dried ice cream samples were extracted in 10 mL of 70% methanol, the supernatant was collected, and the volume was adjusted up to 10 mL with the extraction solvent. An equal volume of Folin–Ciocalteu reagent (1:10) and aliquots of clear supernatant had been mixed and incubated for 5 min before the reaction was stopped by adding Na_2_CO_3_ (7.5%). The TPC content was calculated as milligrams of gallic acid equivalent (GAE) per gram on the basis of an OD reading at 765 nm taken 60 min after incubation, and compared to a standard curve derived from a gallic acid (GA) solution (R^2^ = 0.99) (mg of GAE g^−1^ 100 DW).)

Antioxidant activity (AOA) was assessed using a DPPH^●^ assay [44], and spectrophotometric measurements of the bleaching of DPPH radicals in a purple solution were used to determine the radical scavenging activity (DPPH-RSA). In brief, 1 gram of freeze-dried ice cream sample was extracted in 10 mL of 70% methanol, the supernatant was collected, and the volume was adjusted up to 10 mL with the extraction solvent. A 0.1 mL aliquot from the clear supernatant was mixed with 2.9 mL of DPPH solution and kept in the dark for 60 min. The absorbance was measured at 517 nm. The absorbance change was recorded at 515 nm and then converted into mg of Trolox equivalents (TEs) per gram of dry weight (mg T.E. g^−1^ 100 DW).

According to Mohdaly et al. [45], the TF content of ice cream samples was determined using the same methanolic extract; 1 mL of clear extract aliquots were combined with 1 mL of 2% AlCl_3_ and monitored for 60 min at 420 nm. The data were then expressed as mg quercetin equivalent (Q.E.) per 100 g^−1^ D.W. According to Kumaran and Karunakaran [46], TFL content was determined. Briefly, TFL concentrations were calculated by reacting the methanolic extract aliquots with sodium acetate (5%). AlCl_3_ (2%) was added after 5 min, and the optical density (OD) was measured after 150 min at 440 nm; TFL concentrations are reported in mg QE g^−1^.

#### 2.3.6. Overrun and Melting Behavior

The overrun of different ice cream samples and their melting rate over 120 min at room temperature were measured. To measure the overrun %, the weight of freshly prepared and unfrozen ice cream mix was compared with the weight of the ice cream itself [44], using the following formula:

(1) $$\mathrm{Overrun}\;(\%)=\frac{\mathrm{weight}\;\mathrm{of}\;\mathrm{ice}\;\mathrm{cream}\;\mathrm{mix}-\mathrm{weight}\;\mathrm{of}\;\mathrm{ice}\;\mathrm{cream}}{\mathrm{weight}\;\mathrm{of}\;\mathrm{ice}\;\mathrm{cream}}\;\times \;100$$

The melting point and melting resistance of ice cream samples were measured by melting 50 g at 22 ± 1 °C. Drippings from the samples were collected in a beaker after they had been set on a fine wire screen over a glass funnel. The temperature at which the initial decrease occurred was noted. Drainage weight was measured periodically for a total of 120 min. Melting resistance was determined by calculating the percentage of relative melted amount for each time interval [47].

#### 2.3.7. Instrumental Color Measurements

The color of each sample was measured with a chromameter (ColorFlex, Reston, AV, USA) by applying the CIELAB scale (L*, a*, and b*) adjusted with typical white, green, and black tiles. The hue angle (Hᵒ), chroma (C), and color changes (∆E), and browning index (BI), compared with the values of the control, were then calculated according to Lavelli et al. [48] using continuity Equations (2–5):

(2) $${C=(a}^{2}{+b}^{2}{)}^{0.5}$$

(3) $$H^{{}^{\circ}}{=\tan}^{-1}(\frac{b}{a})$$

(4) $$\mathrm{BI}=\frac{[100\left(\frac{\left(a+1.75L\right)}{\left(5.645L+a-3.012b\right)}\right)-0.31]}{0.17}$$

(5) $$\Delta E^{*}=[{\left(\Delta L\right)}^{2}+{\left(\Delta a\right)}^{2}+{\left(\Delta b\right)}^{2}{]}^{0.5}$$

#### 2.3.8. Rheological Properties

The Discovery Hybrid Rheometer (DHR-2; T.A. Instrument, New Castle, DE, USA) was used to analyze the rheological properties of ice cream samples with different amounts of baobab fruit bulb (ADFP). The applied method conditions were according to the description of Gouda et al. [49]. Briefly, the flow behavior and rheological measurements of ice cream samples after 60 min at 5 °C were examined with the conduction of a parallel geometry sensor (60 nm diameter, 2 cone plate, Peltier plate Aluminum) and 1 mm gap. The function of the shear rate from 0.1 to 100 s^−1^ was obtained for measuring viscosity. The time was adjusted to 120 s during the experiment. In a linear distribution, 32 points were collected, and duplicates had less than 10% differences. Trios software version 3.1.0.3538 was fitted to the power law models for analyzing the data.

#### 2.3.9. Microstructural Examination

The microstructures of ice cream samples were examined at the Electron Microscope Unit, National Research Center, Giza, Egypt. TEM (JEOL, JEM-1230, Charlottesville, VA, USA) was used to investigate the microstructure of freeze-dried different ice cream samples. Samples were prepared according to the technique of Caroll et al. [50], and micrographs (20 Kx) were taken.

#### 2.3.10. Organoleptic Attributes

The organoleptic attributes of the ice cream of flavor (45), body and texture (35), melting quality (10), color and appearance (10), and total score (100) were judged by a panel of 15 staff members at the Department of Food Science and Human Nutrition, Faculty of Agriculture, Qassim University, KSA. Various ice cream samples were coded using random 3-digit numbers and served randomly by the panelists. The panelists were asked to fill in the scorecards, and data were collected for statistical analysis [9].

#### 2.3.11. Statistical Analysis

The statistical analysis was carried out using one-way analysis of variance (ANOVA) using SPSS, v. 22 (IBM Corp. Armonk, NY, USA, Released 2013). Data were treated as a complete randomization design, according to Steel et al. [51]. Multiple comparisons were carried out by applying the Duncan test, the significance level was set at <0.05, and means ± SD were presented.

## 3. Results

### 3.1. Chemical Composition, pH, and Titratable Acidity (TA%)

**Figure 1:**
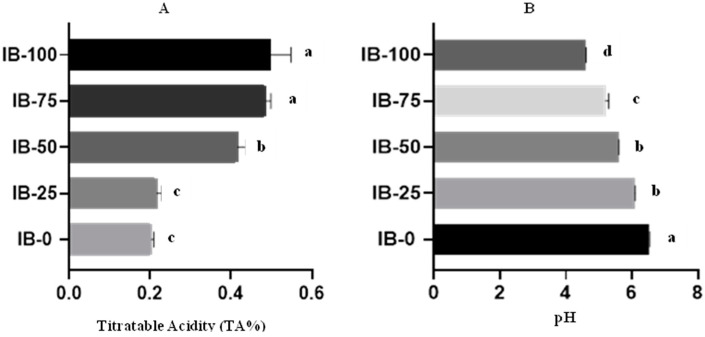
(**A**) Titratable acidity % (TA) and (**B**) pH values of ice cream incorporating ADFP, IB-0: control ice cream, IB-25: ice cream with 25% of its skimmed milk content partially replaced with ADFP, IB-50: ice cream with 50% of its skimmed milk content partially replaced with ADFP, IB-75: ice cream with 75% of its skimmed milk content partially replaced with ADFP, IB-100: ice cream with its skimmed milk content replaced with ADFP. ^a,b,c,d^: Bars not sharing similar letters are statistically different at *p* < 0.05.

The chemical composition of the different ice cream samples (IB-0, IB-25, IB-50, IB-75, and IB-100) is presented in Table 2. No significant differences were observed in the formulas for moisture, TS, fat, and carbohydrates. A significant decrease in TP and Ash% was detected by increasing the amount of ADFP in the ice cream samples. As baobab concentration increased, the pH of the ice cream samples reduced dramatically. ADFP-free sample IB-0 had the highest pH value (6.5) and the lowest TA percentage (0.2%), while the 100% skim milk-replaced sample (IB-100) had the lowest pH value (4.6) and the highest TA (0.51%); pH and TA% are shown in Figure 1.

### 3.2. Microbiological Quality of Different Ice Cream Formulas

The results in Table 3 show the microbiological quality of different ice cream samples and their mixes. The data showed no significant differences in the initial total bacterial counts for ice cream mixes (except for IB-25 and IB-75) or different ice cream samples. In addition, coliform and *Staphylococcus aureus* were not detected in any ice cream samples or their mixes.

### 3.3. Mineral Content

The mineral contents of ice cream samples are presented in Table 4. The results show that the partial substitution with ADFA decreased Na content, but the decrease was not statistically significant. Conversely, the partial substitution of skim milk powder with ADFP in the ice cream samples caused an increase in macroelement amounts, e.g., K (241.5 to 261.2 mg 120 g^−1^) and Mg (1.70 to 2.20 mg 120 g^−1^). An inverse observation was that Ca content significantly decreased from 193.8 to 100.8 mg 120 g^−1^ with the increase in the levels of ADFP. The increase in the Mg content of the ice cream samples from 1.70 to 2.20 mg 120 g^−1^ due to the rise in ADFA levels was insignificant. The contents of microelements such as P (0.62 to 1.12 μg 120 g^−1^), Fe (2.4 to 102.18 μg 120 g^−1^), and Zn (.06 to 18.62 μg120 g^−1^) of the ice cream samples were increased significantly with skim milk powder substitution.

According to Food and Drug Administration (FDA) updates (FDA, 2021), the nutrients’ daily values (DV) are used to inform consumers of the recommended amounts of nutrients that, whether macro- or micronutrients, should be consumed during the day and not exceeded. The daily value (%DV), on the other hand, is the amount of the contribution or content of the nutrient in one serving. Hence, nutritional information should be labeled on different ice cream formulas with the updated daily value for macro- and micronutrients. Table 4. presents the result of macro- and micromineral elements of varying ice cream formulas. The daily values (%DV) for Ca content were increased in IB-0 by 14.9% from the serving daily for calcium. Generally increased values of magnesium, phosphorus, iron, and zinc were found in Sample IB-100 using ADFA at the highest replacement level in the ice cream. The greatest %DV for sodium was in the IB-0 sample.

### 3.4. Total Phenolic Content, Antioxidant Activity, Total Flavonoids (TF), and Total Flavonols (TFL)

The TPC, relative AOA using DPPH, total flavonoids, and total flavonols of all formulated ice cream incorporated ADFP were investigated; data are shown in Table 5. The TPC and AOA were significantly increased with increasing ADFP substitution level, elevating the content of TPC and relevant AOA. Accordingly, the TF and TFL increased with increasing substitution level, and the highest values were recorded when ADFP entirely replaced skim milk powder.

### 3.5. Overrun and Melting Behavior

The highest overrun value was observed for the IB-0 sample (35.61), while the lowest was for the IB-50 (13.71) sample. The melting point was determined via the time required for the first drop of the mixture to decrease to a temperature of 22 °C. The fastest sample dropping during melting was IB-75, which took 35.04 min for the first drop to descend, while the longest time was for the IB-25 sample, where the first drop fell after 44.07 min. The melting resistance of ice cream samples containing ADFP is presented in Table 6. The weight loss % of IB-25 ice cream samples after 45 min at 22 °C was significantly higher than those of all other ice cream samples. In comparison, the most significant loss was after 90 min for the IB-0 samples, which contained no ADFP. A slight increase in the melting point of the ice cream as the inclusion level of ADFP increased was also shown (Table 6), indicating a higher increase in melting resistance up to 120 min with increasing the replacement level of skim milk powder with ADFP.

### 3.6. Instrumental Color Measurements

Color is an essential factor in determining product quality; therefore, it is considered to be a criterion for acceptance by consumers [52]. The results of the color measurement of ice cream incorporating ADFP are presented in Table 7. L* values represent the whitening of the ice cream samples’ color significantly decreasing as the replacement of skim milk powder with ADFP increased and vice versa. Correspondingly, ice cream samples were redder and less green with increasing ADFP amounts, as apparent through an increase in a* and b* values that reflect that the increase in the level of skim milk powder replacement with ADFP in the ice cream samples led to more yellowish and less bluish samples. H^o^ and C values increased significantly with the increase in ADFP levels, and the highest values of BI and ∆E were when replacing 100% of the skim milk powder content with ADFP (33.35 and 83.30, respectively).

### 3.7. Rheological Properties

**Figure 2:**
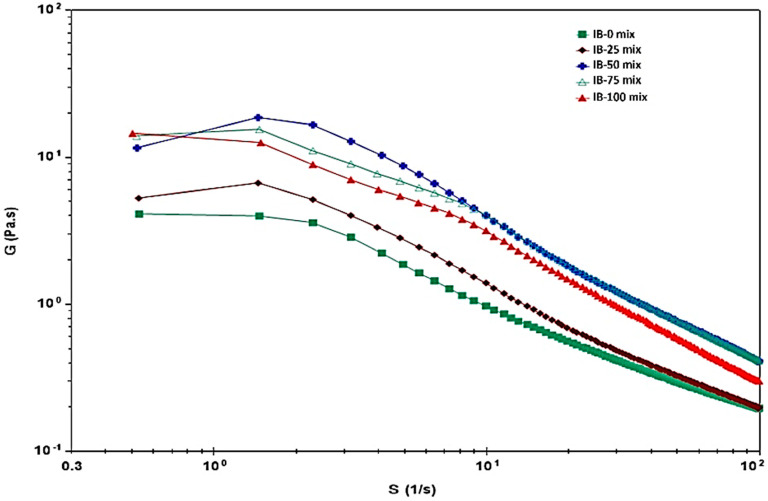
Changes in viscosity as affected by the shear rate of ice cream incorporating ADFP. IB-0: control ice cream, IB-25: ice cream with 25% of its skimmed milk content partially replaced with ADFP, IB-50: ice cream with 50% of its skimmed milk content partially replaced with ADFP, IB-75: ice cream with 75% of its skimmed milk content partially replaced with ADFP, IB-100: ice cream with its skimmed milk content replaced with ADFP.

As a function of shear rate, the viscosity of various ice cream samples is represented in Figure 2, and their rheological parameters (η_0_: zero-rate viscosity, η_00_: infinite-rate viscosity, K: consistency, n: rate index) are shown in Table 8. Since ice cream is a shear-thinning non-Newtonian fluid with pseudoplastic flow, the apparent viscosity for all ice cream samples was decreased with the increase in shear rate, showing pseudoplastic behavior. In the entire shear rate range (100–101), the highest viscosity was for the IB-50 ice cream sample, while the lowest was for the IB-0 sample. As a result of skim milk powder replacement with ADFP, all samples (IB-25, IB-50, IB-75, and IB-100) had a higher viscosity than that of the ADFP-free sample (IB-0). Otherwise, the level of ADFP replacement had a reversible effect on viscosity when the replacement percentage reached 75 and 100% in IB-75 and IB-100, respectively, among samples containing ADFP.

The replacement of skim milk powder with ADFP significantly increased the infinite rate viscosity (η_00_) compared to that of the IB-0 sample, which contained no ADFP. The more the ADFP amount increased, the greater the viscosity (Pa·s) that was observed in all samples (IB-25: 0.11, IB-50:0.17, and IB-75:0.18) except for IB-100 with zero skim milk powder. The replacement of 75% skim milk with ADFP (sample: IB-75) significantly increased viscosity by about 35.5% more than that of the IB-0 sample, with no significant difference between IB-50 and IB-75 in infinite rate viscosity. The increase in ADFP with the absence of skim milk powder (IB-100) markedly decreased viscosity by about 23.1% more than that of the IB-0 sample with 100% skim milk powder content.

The consistency coefficient (K) of the ice creams indicating their relative thickness increased with the presence of ADFP in Samples IB-25 (0.875 Pa·sn) and IB-50 (0.498 Pa·sn), while the opposite trend was observed in the rest of the samples. No significant (*p* > 0.05) differences were observed in K values between IB-75 and IB-100 compared to the IB-0 sample. An increase in rate index was significantly observed in samples with greater ADFP amounts (IB-75 and IB-100) than those of the rest of the samples.

### 3.8. Microstructural Examination

**Figure 3:**
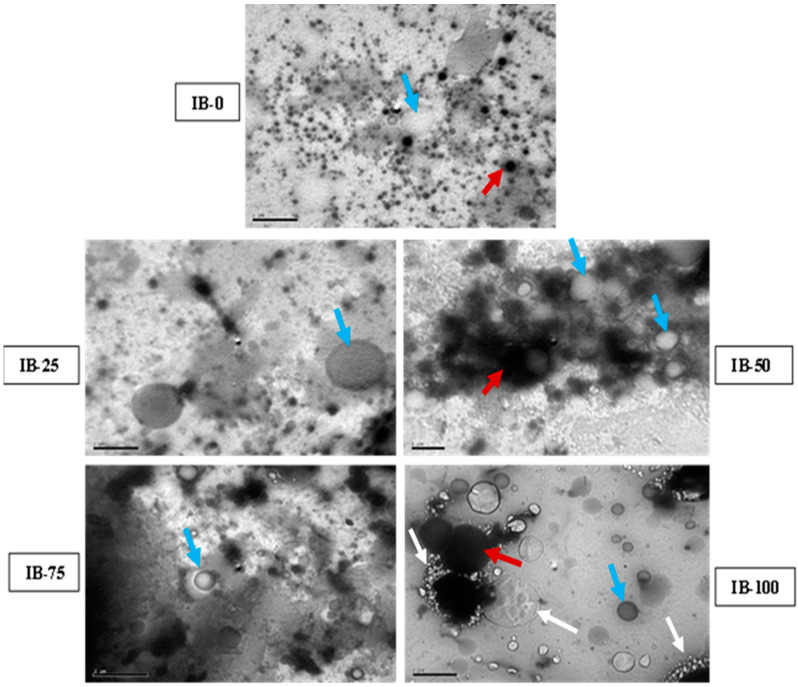
Transmission electron micrographs (1 μm bar) of ice cream samples in ice cream incorporating ADFP. Protein clusters (red arrow), fat globules (blue arrow), and water separation (white arrow). IB-0: control ice cream, IB-25: ice cream with 25% of its skimmed milk content partially replaced with ADFP, IB-50: ice cream with 50% of its skimmed milk content partially replaced with ADFP, IB-75: ice cream with 75% of its skimmed milk content partially replaced with ADFP, IB-100: ice cream with its skimmed milk content replaced with ADFP.

The transmission electron micrographs of different ice cream samples are presented in Figure 3. Protein–protein and protein–fat interactions, fat globules, casein micelles, and phase interfaces are distinguished in different fields of electron microscopy observed in the current study. An electron micrograph of the IB-0 ice cream sample, which contained no ADFP, showed small fat globules (blue arrow) and protein particles (red arrow) with uniform distribution. The most distinguishing features in that micrograph were the apparent quantity of nonaggregated micellar material surrounding the fat globules. The interfacial adsorption of protein particles, mainly casein micelles, to fat globules could be seen with no evident signs of merged casein micelles or pronounced aggregation in this sample. Those observations typically represent the normal distribution of ice cream phases under TEM. Previous observations were changed in the micrographs of the remaining samples (IB-25, IB-50, and IB-75). The more attractive observation in those fields was that fat globules were coated with thick layers of aggregated protein. Casein particles also lost their regular spherical deformation and collapsed together in large aggregates, leaving a wide area of serum phase separation. Unique shapes appeared in the TEM micrograph of Sample IB-100 (100% of skim milk powder replaced with ADFP). Large protein agglomerates were surrounded by shiny white aggregates of differing sizes, whereas the fat globules appeared independently without any protein surrounding them.

### 3.9. Organolyptical Attributes

**Figure 4:**
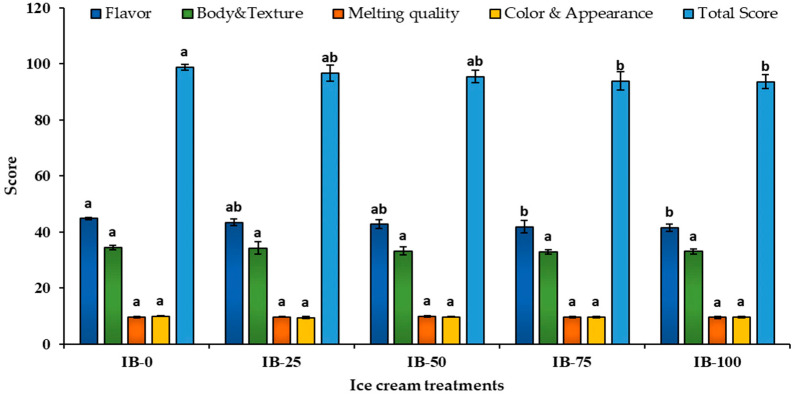
Organoleptic attributes scores of ice cream incorporating ADFP. IB-0: control ice cream, IB-25: ice cream with 25% of its skimmed milk content partially replaced with ADFP, IB-50: ice cream with 50% of its skimmed milk content partially replaced with ADFP, IB-75: ice cream with 75% of its skimmed milk content partially replaced with ADFP, IB-100: ice cream with its skimmed milk content replaced with ADFP. ^a,b^: Bars not sharing similar letters are statistically different at *p* < 0.05.

The mean scores of the flavor, texture, melting quality, and appearance of the different ice cream samples as affected by ADFP addition are presented in Figure 4. The ranges of the organoleptic scores among the ice cream samples were: flavor, 41.50–44.80; body and taste, 32.90–34.50; melting quality, 9.50–9.80; appearance, 9.40–9.90. No significant differences (*p* > 0.05) were found among all ice cream samples regarding body and texture, melting quality or color, and appearance, as reported by the panelists. The difference among samples with only the scores of flavors changed the total scores. The flavor attribute for the IB-100 sample (*p* ˂ 0.05) significantly had the lowest score among all other samples. The main remarks by the panelists were that the IB-75 and IB-100 samples lacked smoothness, and had grainy and rough structures.

## 4. Discussion

This study is closely related to that reported by Umelo et al. [53] on tiger nut ice cream. Baobab fruit pulp supplied a quantity of soluble (22.54% dry weight) and insoluble (22.04% dry weight) fibers [54]. That increase with no significance in carbohydrate concentrations in different samples compared with the IB-0 sample may have been because fiber content increases the apparent amount of digestible carbohydrates because carbohydrate is calculated via the mass remaining after protein, fat, ash, and fibers are subtracted from 100 g of the sample [55]. Those are attributed to the presence of organic acids in baobab powder, including citric, tartaric, malic, succinic, and ascorbic acid, as mentioned by Ahmed et al. [56]. For this reason, acidity content increased as baobab increased from 0.2% at 0% powder to 0.5% at 10%. Dabora found that yogurt acidity increases as baobab fruit pulp powder increases [57].

According to most hygiene guidelines (i.e., EEC 1992; E.C. 1999; NZICMA 2002), TBC should be 0–10^5^ CFUg^−1^ or less. Many processing steps after the pasteurization of ice cream render it vulnerable to bacterial contamination, especially from pathogenic microbes that contaminate the product after its heat treatment. The microbiological quality of ice cream depends on many factors, including the microbial standards of the raw materials, the production process, and the hygiene of the working environments [58]. Cooling the liquid mix, which is kept at 4 °C for a few hours up to a maximum of 24 h, favors its aging. Maintaining suitable temperatures during the cooling step limits the growth of bacteria that have combated pasteurization or recontaminated the mixture after heat treatment. The air responsible for the typical soft texture of the ice cream can also be involved in recontamination, and the final containers in the ice cream are distributed [59]. The obtained results show that the ice cream samples were within acceptable microbiological limits because TBC was <10^5^ CFU/g and pathogenic bacteria were absent. This reflects the hygiene and quality of the used materials, and the preparation and production processes of the ice cream under study. Ice cream is a complex food model, and comprehensive approaches to the entire production process are required to ensure high quality and safety standards.

Incorporating ADFP into food can be a crucial source of essential nutrients for food fortification due to its high content of macro- and microelements [14,36] results in Table 4 show that K and Mg were the most abundant macroelements, while Na content was low. Similar results were observed in previous studies [14,17]. Magnesium plays a vital role in bone metabolism. Its deficiency is a possible risk factor for osteoporosis, while potassium is essential to the human body [60]. Results also show that Na content was low compared to that of K, suggesting that a high K/Na ratio can protect from high blood pressure [61]. Our findings indicate that all ice cream samples incorporating ADFP led to significant P, Zn, and Fe content increments, improving the product’s nutritional status [62]. The distributions of macro- and microelements in different ice cream samples could have been due to the content of elements in both skim milk powder and ADFP, and their relative amounts in each ice cream sample. The results obtained by Osman [14] and Barakat [63] showed that ADFP is an excellent source of sodium (29.7–36.0 mg 100 g^−1^), potassium (988–1240 mg 100 g^−1^), calcium (237–295 mg 100 g^−1^), magnesium (90–110 mg 100 g^−1^), and phosphorus (124 mg 100 g^−1^), but poor sources of iron, zinc, and copper. On the other hand, skim milk powder is also a good source of sodium (470 mg 100 g^−1^), potassium (1490 mg 100 g^−1^), calcium (1140 mg 100 g^−1^), magnesium (110 mg 100 g^−1^), and phosphorus (500 mg 100 g^−1^), as reported by Arellano et al. [64]. From the ongoing results, it could be logically reported that the amounts of calcium should be decreased. In contrast, the amount of potassium and phosphorus should be increased, as the amount of replacement with ADFP increases and vice versa.

Interestingly, a remarkable incremental trend in TPC, AOA, T.F., and TFL in formulated ice cream incorporating BPF was observed. This may have been due to the rich content of bioactive compounds and phytochemicals in BPF, which was increased with increasing ADFP levels. The substitution of skimmed milk powder with BPF in ice cream formulas slightly improved the AOA assessed with DPPH scavenging assays. The antioxidant capacity was probably due to the presence of phenolic compounds and ascorbic acid in ADFP [44,63]. The AOA could improve ice cream shelf-life stability and delay fat oxidation [44,63].

The increasing ADFP level increased the melt resistance of the prepared ice cream. This improvement was due to the high foaming stability of ADFP and good formulated structure seen in TEM structural analysis (Figure 3). The combination of ADFP and added ice cream ingredients improved structure, foam stability, conserved air bubbles, and accelerates fat globules and protein aggregates with unfrozen serum retention comprising its complex structure [58,63]. This could be argued because foamy substances occurred in ADFP [24,25].

The lack of creaminess and smoothness came from the extremely low overrun, indicating that little air was included, causing an excessively cold sensation in the mouth. On the other hand, a high overrun amount makes the ice cream frothy, even though some countries set 100% as the legal overrun limit for ice cream [65]. Numerous factors, such as ice crystal size, content, overrun, the extent of fat destabilization, and the rheological properties of the mixtures, influence the hardness of ice cream [44,66].

The color of ice cream is a crucial sensorial parameter that reflects its consumers’ acceptability. The formulation of an ice cream mixture and the coloring ability of the product’s fruit affect the final product’s color quality [67]. As previously reported, red, green, blue, and yellow are colors defined by hue (h), lightness (L*) is the parameter for color brightness, and chromaticity (C) or colorfulness represents the color sensation; all are measured using instrumental colorimeters [68]. The effect of incorporating ADFP into ice cream samples positively affected their color (Table 6). The significant decrease in lightness (L*) and increase in redness (a*) and yellowness (b*) with increasing ADFP replacement amounts of skim milk powder from 0% to 100% indicated that ADFP could be used as a natural coloring agent to produce colored ice cream (off-white to brown) depending on its concentration level. The values of BI and ∆E increased associatively when skim milk was replaced, which presented a desired change in the prepared ice cream. For instance, adding more ADFP would improve the color; however, seeking ideal physical and rheological characteristics is something that cannot be overlooked. These color changes are combined with the ADFP color, described as light brown to reddish brown, depending on the fruit type [69].

In this study, zero- and infinite-rate viscosity values, as a relation to the shear rate in ice cream samples with opposite amounts of skim milk powder and ADFP, were near the ranges of 4.49 to 15.88 and 0.13 to 0.18 Pa·s, respectively, as the amount of ADFP increased, except for the IB-100 sample. The effect of increasing viscosity as the ADFP amounts increased was also related to the increase in TA% and decrease in pH values (Figure 1), and the decline in the overrun % for all ice cream samples. Moreover, results obtained by Barros et al. [70] indicated that the addition of concentrated whey in ice cream formulation markedly increased the consistency of ice cream samples with an increase in protein content which tends to increase consistency index and viscosity, and consequently, increased the viscosity of ice creams. They concluded that, because of the attribution of the large dispersion of denatured protein colloidal particles and casein micelles in the structure of ice cream, different protein sources would impact consistency as protein content increased. As with denatured whey proteins, increasing TA% and decreasing pH values may have the same effect as agglomerating proteins in large aggregates, which leads to the excess thickening and gelation of an ice cream mix, resulting in inverse melting behavior with both viscosity and consistency.

Observed increments in zero-rate viscosity, infinite-rate viscosity, and consistency as a result of the ADFP replacement increase (Table 7) were found in our study. A negative effect on the flow of the melting behavior of the ADFP-containing samples (IB-25, IB-50, IB-75, and IB-100), as presented in Table 6, was also found. This relationship was previously explained by Amador et al. [71], who mentioned that, as viscosity increased, resistance to flow increased, which slowed the drip-through pace through the mesh screen.

The texture properties of food are those perceptions based on the evaluation of its physical characteristics as affected by parameters related to the shape, size, structure, number, and conformation of its constituent structural elements and proteins. All play a crucial role in forming the texture of the final product. The protein–fat matrix in an ice cream structure and a cohesive network are essential to produce a homogeneous fat–liquid emulsion. Thus, the combination of many ingredients in the formulation of ice cream, and the four systems that comprise its structure, each an emulsion and each a foam, ice crystals, air bubbles, fat globules, aggregates, and unfrozen serum, compose its complex structure. Thus, TEM is recommended to observe the ice cream microstructure [58,63,72,73,74].

As compared to the IB-0 sample, ice cream samples containing ADFP showed apparent differences in electron micrographs (Figure 3). Protein deformation and aggregation were associated with a reduction in pH values (Figure 1). As the amount of ADFP replacement increased, these changes and phase separation in the microstructure became more evident, especially in the IB-100 sample with a 100% replacement rate. This observation was in agreement with Karlsson and Ardö [75]. They mentioned that, at pH 5.8, when preparing skim milk powder for TEM examination, the borders of the casein micelles diffused, signs of a merger between micelles, and a loss of micellar individuality appeared. In addition, Mckenna et al. [76] showed that using TEM to observe whole milk powder microstructure, aggregation, and the association of lactoglobulin with casein microspheres increased as the pH of milk decreased. So, the low pH contributed to increased whey protein aggregation, and improved interactions between casein micelles in dairy products containing milk powders such as ice cream, which may lead to liquid-phase separation.

In both IB-75 and IB-100, the significant decrease in flavor significantly decreased the total score for these samples. This result was in agreement with Ali and El Zubeir [77], who found that, when adding 3 and 5% of baobab as a flavoring substance to a camel-milk-based ice cream mix, the flavor score of the final product decreased compared to the vanilla ice cream sample. A decline in flavor score may be due to the acid-tangy, tart flavor of the baobab fruit pulp [12,13,14].

## 5. Conclusions

The effect of replacing skim milk powder with *Adansonia digitata* L. fruit pulp (ADFP) on the physicochemical, nutritional, rheological, microstructure, and organoleptic properties of ice creams was investigated. Incorporating ADFP into ice cream improved dietary values regarding macro- and micronutrients. Additionally, antioxidant and mineral content enhancement was also detected, which could enhance the ice cream’s stability. Ice cream with no more than 75% ADFP instead of skim milk powder improved the microstructural and rheological properties. When the ADFP level increased, ice cream acceptability was increased regarding organoleptic properties. Remarkably, data gained from the current study highly recommend such a replacement. ADPF can be safely employed as a natural coloring ingredient in ice cream without affecting its microstructural or rheological properties. It also plays a crucial role in improving the ice cream’s nutritional value.

## Figures and Tables

**Table 1:** Ingredients of different ice cream formulations incorporating ADFP.

Formula	Ingredients (g 100 g^−1^)						
	Cow Milk (3% Fat)	Cream (55% Fat)	Skim Milk (95%SNF)	Sugar	Gelatin	ADFP	Total
IB-0	64.8	17.9	4.8	12	0.5	0	100
IB-25	64.8	17.9	3.6	12	0.5	1.2	100
IB-50	64.8	17.9	2.4	12	0.5	2.4	100
IB-75	64.8	17.9	1.2	12	0.5	3.6	100
IB-100	64.8	17.9	0	12	0.5	4.8	100

IB-0: control ice cream, IB-25: ice cream with 25% of its skimmed milk content partially replaced with ADFP, IB-50: ice cream with 50% of its skimmed milk content partially replaced with ADFP, IB-75: ice cream with 75% of its skimmed milk content partially replaced with ADFP, IB-100: ice cream with its skimmed milk content replaced with ADFP.

**Table 2:** The proximate chemical composition of ice cream incorporating ADFP.

Treatment	Chemical Composition (g 100 g^−1^)					
	Moisture	TS	TP	Fat	Ash	Carbohydrates
IB-0	64.00 ± 0.73 ^a^	36.00 ± 0.73 ^a^	4.70 ± 0.57 ^a^	11.87 ± 0.06 ^a^	0.89 ± 0.05 ^a^	18.56 ± 0.56 ^a^
IB-25	64.12 ± 0.42 ^a^	35.94 ± 0.42 ^a^	4.14 ± 0.27 ^ab^	11.83 ± 0.25 ^a^	0.74 ± 0.05 ^bc^	19.17 ± 0.03 ^a^
IB-50	64..06 ± 0.73 ^a^	35.94 ± 0.73 ^a^	4.13 ± 0.23 ^ab^	11.83 ± 0.32 ^a^	0.74 ± 0.02 ^bc^	19.24 ± 0.87 ^a^
IB-75	64.14 ± 0.15 ^a^	35.86 ± 0.15 ^a^	3.95 ± 0.16 ^b^	11.87 ± 0.25 ^a^	0.76 ± 0.05 ^b^	19.30 ± 0.47 ^a^
IB-100	64.19 ± 0.66 ^a^	35.81 ± 0.67 ^a^	3.93 ± 0.19 ^b^	11.83 ± 0.32 ^a^	0.68 ± 0.01 ^c^	19.37 ± 1.13 ^a^

TS: total solids, TP: total crude protein (TNx6.38), IB-0: control ice cream, IB-25: ice cream with 25% of its skimmed milk content partially replaced with ADFP, IB-50: ice cream with 50% of its skimmed milk content partially replaced with ADFP, IB-75: ice cream with 75% of its skimmed milk content partially replaced with ADFP, IB-100: ice cream with its skimmed milk content replaced with ADFP. ^a,b,c^: There is no significant difference (*p* > 0.05) between any two means, within the same column have the same superscripted letters.

**Table 3:** Viable bacterial counts (log CFU g^−1^) in ice cream incorporating ADFP.

Sample Type	Sample				
	IB-0	IB-25	IB-50	IB-75	IB-100
Ice cream mix	2.89 ± 0.11 ^b^	3.35 ± 0.05 ^a^	2.76 ± 0.15 ^b^	2.39 ± 0.09 ^c^	2.73 ± 0.12 ^b^
Ice cream sample	2.73 ± 0.58 ^b^	3.02 ± 0.08 ^b^	2.76 ± 0.06 ^b^	2.90 ± 0.10 ^ab^	2.77 ± 0.15 ^b^

Log CFU: log of colony-forming units, IB-0: control ice cream, IB-25: ice cream with 25% of its skimmed milk content partially replaced with ADFP, IB-50: ice cream with 50% of its skimmed milk content partially replaced with ADFP, IB-75: ice cream with 75% of its skimmed milk content partially replaced with ADFP, IB-100: ice cream with its skimmed milk content replaced with ADFP. ^a,b,c^: There is no significant difference (*p* > 0.05) between any two means, within the same column have the same superscripted letters.

**Table 4:** Macro- and microelements in different samples represented as amounts per one ice cream serving (120 g).

Ice Cream	Macroelements * (mg 120 g^−1^)					Microelements ** (μg120 g^−1^)	
	Na	K	Ca	Mg	*p*	Fe	Zn
	Elemental amount						
IB-0	144.50 ± 58.1 ^a^	241.50 ± 25.5 ^a^	193.80 ± 18.6 ^a^	1.70 ± 1.2 ^a^	0.62 ± 0.0 ^e^	<2.4 ± 0.0 ^c^	0.06 ± 0.0 ^e^
IB-25	143.50 ± 57.1 ^a^	247.80 ± 11.4 ^a^	190.80 ± 8.4 ^a^	1.71 ± 1.4 ^a^	0.73 ± 0.0 ^d^	<2.4 ± 0.0 ^c^	2.75 ± 0.0 ^d^
IB-50	115.30 ± 57.7 ^a^	254.40 ± 1.8 ^a^	156.00 ± 1.2 ^b^	1.72 ± 1.0 ^a^	0.76 ± 0.0 ^c^	<2.4 ± 0.0 ^c^	11.34 ± 0.3 ^c^
IB-75	115.30 ± 57.7 ^a^	259.80 ± 13.8 ^a^	141.00 ± 19.8 ^b^	1.82 ± 0.9 ^a^	1.07 ± 0.0 ^b^	79.32 ± 0.7 ^b^	14.64 ± 0.3 ^b^
IB-100	83.90 ± 5.5 ^a^	261.60 ± 12.0 ^a^	100.80 ± 19.2 ^c^	2.20 ± 0.2 ^a^	1.12 ± 0.0 ^a^	102.18 ± 2.5 ^a^	15.62 ± 0.1 ^a^
	Elements contributing to a daily diet (%DV)						
IB-0	6.28 ± 2.53 ^a^	5.14± 0.54 ^a^	14.91 ± 1.43 ^a^	0.40 ± 0.29 ^a^	0.049± 0.00 ^e^	<0.013 ± 0.00 ^c^	0.0005 ± 0.00 ^e^
IB-25	6.24 ± 2.48 ^a^	5.27 ±0.24 ^a^	14.68 ± 0.65 ^a^	0.41 ± 0.33 ^a^	0.059 ± 0.00 ^d^	<0.013 ± 0.00 ^c^	0.02 ± 0.00 ^d^
IB-50	5.01 ± 2.51 ^a^	5.48 ±0.04 ^a^	12.00 ± 0.09 ^b^	0.41 ± 0.24 ^a^	0.061 ± 0.00 ^c^	<0.013 ± 0.00 ^c^	0.10 ± 0.00 ^c^
IB-75	4.64 ± 2.14 ^a^	5.53 ±0.29 ^a^	10.85 ± 1.52 ^b^	0.43 ± 0.21 ^a^	0.086 ± 0.00 ^b^	0.441 ± 0.01 ^b^	0.13 ± 0.00 ^b^
IB-100	3.65 ± 240 ^a^	5.57 ±0.26 ^a^	7.75 ± 1.48 ^c^	0.52 ± 0.05 ^a^	0.089± 0.00 ^a^	0.584 ± 0.137 ^a^	0.14 ± 0.00 ^a^

IB-0: control ice cream, IB-25: ice cream with 25% of its skimmed milk content partially replaced with ADFP, IB-50: ice cream with 50% of its skimmed milk content partially replaced with ADFP, IB-75: ice cream with 75% of its skimmed milk content partially replaced with ADFP, IB-100: ice cream with its skimmed milk content replaced with ADFP; *: DV of macroelements (mg): Na (sodium): 2300, K (potassium): 4700, Ca (calcium): 1300, Mg (magnesium): 420 and P (phosphorus): 1250, **: DV of micromineral elements (μg): Fe (iron): 18000 and Zn (zinc): 11000. ^a,b,c,d,e^: There is no significant difference (*p* > 0.05) between any two means, within the same column have the same superscripted letters.

**Table 5:** Total phenolic content, antioxidant activity total, flavonoids (TF), and total flavanols (TFL) in ice cream incorporating ADFP.

Antioxidant Parameters	Ice Cream Sample				
	IB-0	IB-25	IB-50	IB-75	IB-100
TPC (mg GAE 100 g^−1^)	123.78 ± 1.23 ^e^	127.69 ± 0.98 ^d^	134.90 ± 1.98 ^c^	142.37 ± 2.57 ^b^	149.29 ± 3.18 ^a^
DPPH (µmol of TE 100 g^−1^)	89.17 ± 1.23 ^d^	91.27 ± 0.45 ^d^	94.99 ± 0.71 ^c^	96.24 ± 0.29 ^b^	98.12 ± 0.89 ^a^
TF (mg QE 100 g^−1^)	00 ± 00 ^d^	4.16 ± 0.23 ^c^	4.76 ± 0.14 ^b^	5.06 ± 0.39 ^b^	5.96 ± 0.23 ^a^
TFL (mg QE 100 g^−1^)	00 ± 00 ^d^	2.96 ± 0.27 ^c^	3.28 ± 0.19 ^bc^	3.65 ± 0.21 ^b^	4.01 ± 0.27 ^a^

IB-0: control ice cream, IB-25: ice cream with 25% of its skimmed milk content partially replaced with ADFP, IB-50: ice cream with 50% of its skimmed milk content partially replaced with ADFP, IB-75: ice cream with 75% of its skimmed milk content partially replaced with ADFP, IB-100: ice cream with its skimmed milk content replaced with ADFP; ^a,b,c,d,e^: means with the same superscripted letters in the same row were not significantly different (*p* < 0.05).

**Table 6:** Overrun, melting point, and weight loss (%) in interval times during storage for 120 min at 25 °C of ice cream incorporating ADFP.

Ice Cream Sample	Overrun %	Starting Melting Point (min)	Weight Loss %				
			45 min	75 min	90 min	105 min	120 min
IB-0	35.61 ± 0.53 ^a^	37.57 ± 5.15 ^b^	12.49 ± 0.04 ^b^	89.54 ± 0.31 ^a^	92.17 ± 0.16 ^a^	-	-
IB-25	20.21 ± 0.200 ^b^	35.93 ± 0.08 ^b^	13.37 ± 0.07 ^a^	65.40 ± 0.15 ^b^	89.34 ± 0.11 ^b^	-	-
IB-50	13.71 ± 0.25 ^b^	44.07 ± 0.12 ^a^	0.28 ± 0.03 ^e^	1.42 ± 0.03 ^e^	1.96 ± 0.02 ^e^	45.26 ± 0.05 ^a^	45.30 ± 0.02 ^a^
IB-75	19.39 ± 0.03 ^c^	35.04 ± 0.16 ^b^	1.64 ± 0.01 ^d^	3.65 ± 0.01 ^d^	4.53 ± 0.07 ^d^	9.29 ± 0.02 ^c^	25.16 ± 0.16 ^b^
IB-100	20.1 ± 0.36 ^d^	35.33 ± 0.31 ^b^	2.97 ± 0.21 ^c^	8.22 ± 0.10 ^c^	10.45 ± 0.06 ^c^	12.19 ± 0.12 ^b^	12..63 ± 0.49 ^c^

IB-0: control ice cream, IB-25: ice cream with 25% of its skimmed milk content partially replaced with ADFP, IB-50: ice cream with 50% of its skimmed milk content partially replaced with ADFP, IB-75: ice cream with 75% of its skimmed milk content partially replaced with ADFP, IB-100: ice cream with its skimmed milk content replaced with ADFP; ^a,b,c,d,e^: There is no significant difference (*p* < 0.05) between any two means, within the same column have the same superscripted letters.

**Table 7:** Color changes of ice cream samples of ice cream incorporating ADFP.

Ice Cream Sample	L*	a*	b*	C	H°	BI	∆E
IB-0	85.69 ± 0.09 ^a^	−3.45 ± 0.22 ^d^	6.23 ± 0.14 ^e^	7.12 ± 0.23 ^e^	118.91 ± 1.00 ^a^	4.339 ± 0.18 ^e^	0.00 ± 0.00 ^e^
IB-25	80.22 ± 0.51 ^b^	−2.29 ± 0.20 ^c^	9.23 ± 0.05 ^d^	9.52 ± 0.03 ^d^	103.91 ± 1.22 ^b^	10.13 ± 0.26 ^d^	72.22 ± 0.13 ^d^
IB-50	77.00 ± 0.02 ^c^	−0.02 ± 0.21 ^b^	15.51 ± 0.13 ^c^	15.51 ± 0.13 ^c^	90.04 ± 0.72 ^c^	23.96 ± 0.04 ^c^	75.46 ± 0.04 ^c^
IB-75	73.51 ± 0.03 ^d^	0.67 ± 0.05 ^b^	17.03 ± 0.05 ^b^	17.05 ± 0.05 ^b^	87.79 ± 0.16 ^d^	26.43 ± 0.03 ^b^	78.38 ± 0.02 ^b^
IB−100	71.21 ± 0.11 ^e^	1.47 ± 0.83 ^a^	22.39 ± 0.93 ^a^	22.45 ± 0.92 ^a^	86.27 ± 2.12 ^e^	33.35 ± 1.62 ^a^	83.30 ± 0.25 ^a^

BI: Browning index, IB-0: control ice cream, IB-25: ice cream with 25% of its skimmed milk content partially replaced with ADFP, IB-50: ice cream with 50% of its skimmed milk content partially replaced with ADFP, IB-75: ice cream with 75% of its skimmed milk content partially replaced with ADFP, IB-100: ice cream with its skimmed milk content replaced with ADFP, ^a,b,c,d,e^: means with the same superscripted letters in the same row were not significantly different (*p* < 0.05).

**Table 8:** Rheological properties (mean ± SD) of ice cream incorporating ADFP.

Ice Cream Sample	Power Law Model				
	η_0_ (Pa·s)	η_00_ (Pa·s)	K (Pa·s^n^)	Rate Index	r^2^
IB-0 mix	4.49 ± 1.31 ^c^	0.13 ± 0.01 ^b^	0.337 ± 0.102 ^c^	1.14 ± 0.01 ^b^	0.999 ± 0.00 ^a^
IB-25 mix	15.62 ± 0.63 ^b^	0.11 ± 0.00 ^b^	0.875 ± 0.007 ^a^	1.18 ± 0.04 ^ab^	0.999 ± 0.00 ^a^
IB-50 mix	30.46 ± 8.44 ^a^	0.17 ± 0.02 ^a^	0.498± 0.102 ^b^	1.21 ± 0.02 ^ab^	0.999± 0.00 ^a^
IB-75 mix	18.52 ± 1.18 ^b^	0.18 ± 0.00 ^a^	0.318± 0.032 ^c^	1.31 ± 0.04 ^a^	0.998 ± 0.00 ^a^
IB-100 mix	15.88 ± 2.48 ^b^	0.10 ± 0.03 ^b^	0.350 ± 0.076 ^c^	1.28 ± 0.13 ^a^	0.998 ± 0.00 ^a^

η_0_: zero-rate viscosity, η_00_: infinite-rate viscosity, K: consistency, IB-0: control ice cream, IB-25: ice cream with 25% of its skimmed milk content partially replaced with ADFP, IB-50: ice cream with 50% of its skimmed milk content partially replaced with ADFP, IB-75: ice cream with 75% of its skimmed milk content partially replaced with ADFP, IB-100: ice cream with its skimmed milk content replaced with ADFP; ^a,b,c^: means with the same superscripted letters in the same row were not significantly different (*p* < 0.05).

## Data Availability

Data are contained within the article.

## References

[1] Villava F.J., Cravero Bruneri A.P., Vinderola G., Goncalvez De Oliveira E., PAZ N.F., Ramon A.N. (2017). Formulation of a Peach Ice Cream as Potential Symbiotic Food. Food Sci. Technol..

[2] Thorning T.K., Bertram H.C., Bonjour J.-P., De Groot L., Dupont D., Feeney E., Ipsen R., Lecerf J.M., Mackie A., McKinley M.C. (2017). Whole Dairy Matrix or Single Nutrients in Assessment of Health Effects: Current Evidence and Knowledge Gaps. Am. J. Clin. Nutr..

[3] Astrup A. (2014). Yogurt and Dairy Product Consumption to Prevent Cardiometabolic Diseases: Epidemiologic and Experimental Studies. Am. J. Clin. Nutr..

[4] Elwood P.C., Pickering J.E., Givens D.I., Gallacher J.E. (2010). The Consumption of Milk and Dairy Foods and the Incidence of Vascular Disease and Diabetes: An Overview of the Evidence. Lipids.

[5] Sun-Waterhouse D. (2011). The Development of Fruit-Based Functional Foods Targeting the Health and Wellness Market: A Review. Int. J. Food Sci. Technol..

[6] Van Kleef E., Van Trijp H.C., Luning P., Jongen W.M. (2002). Consumer-Oriented Functional Food Development: How Well Do Functional Disciplines Reflect the ‘Voice of the Consumer’?. Trends Food Sci. Technol..

[7] Cruz A.G., Antunes A.E., Sousa A.L.O., Faria J.A., Saad S.M. (2009). Ice-Cream as A Probiotic Food Carrier. Food Res. Int..

[8] Smith J.S., Hui Y.H. (2008). Food Processing: Principles and Applications.

[9] Arbuckle W.S. (2013). Ice Cream.

[10] Soukoulis C., Lebesi D., Tzia C. (2009). Enrichment of Ice Cream With Dietary Fibre: Effects on Rheological Properties, Ice Crystallisation and Glass Transition Phenomena. Food Chem..

[11] Tiwari A., Sharma H.K., Kumar N., Kaur M. (2015). The Effect of Inulin as A Fat Replacer on the Quality of Low-Fat Ice Cream. Int. J. Dairy Technol..

[12] Rahul J., Jain M.K., Singh S.P., Kamal R.K., Anuradha, Naz A., Gupta A.K., Mrityunjay S.K. (2015). *Adansonia digitata* L. (Baobab): A Review of Traditional Information and Taxonomic Description. Asian Pac. J. Trop. Biomed..

[13] Kaboré D., Sawadogo-Lingani H., Diawara B., Compaoré C.S., Dicko M.H., Jakobsen M. (2011). A Review of Baobab (*Adansonia digitata*) Products: Effect of Processing Techniques, Medicinal Properties and Uses. Afr. J. Food Sci..

[14] Osman M.A. (2004). Chemical and Nutrient Analysis of Baobab (*Adansonia digitata*) Fruit and Seed Protein Solubility. Plant Foods Hum. Nutr..

[15] Yazzie D., VanderJagt D.J., Pastuszyn A., Okolo A., Glew R.H. (1994). The Amino Acid and Mineral Content of Baobab (*Adansonia digitata* L.) Leaves. J. Food Compos. Anal..

[16] De Caluwé E., Halamová K., Van Damme P. (2010). *Adansonia digitata* L. A Review of Traditional Uses, Phytochemistry and Pharmacology. Afr. Focus.

[17] Muthai K.U., Karori M.S., Muchugi A., Indieka A.S., Dembele C., Mng’omba S., Jamnadass R. (2017). Nutritional Variation in Baobab (*Adansonia digitata* L.) Fruit Pulp and Seeds Based on Africa Geographical Regions. Food Sci. Nutr..

[18] Hyacinthe T., Charles P., Adama K., Diarra C.S., Dicko M.H., Svejgaard J.J., Diawara B. (2015). Variability of Vitamins B1, B2 and Minerals Content in Baobab (*Adansonia digitata*) Leaves in East and West Africa. Food Sci. Nutr..

[19] Monteiro S., Reboredo F.H., Lageiro M.M., Lourenço V.M., Dias J., Lidon F., Abreu M., Martins A.P.L., Alvarenga N. (2022). Nutritional Properties of Baobab Pulp from Different Angolan Origins. Plants.

[20] Busson F., Deniel P., Toury J. (1958). Amino Acid Composition of the Leaves and Fruit Pulp of Baobab (*Adansonia digitata* Linn.). Bull. Soc. Chim. Biol..

[21] Li X.N., Sun J., Shi H., Yu L.L., Ridge C.D., Mazzola E.P., Okunji C., Iwu M.M., Michel T.K., Chen P. (2017). Profiling Hydroxycinnamic Acid Glycosides, Iridoid Glycosides, and Phenylethanoid Glycosides in Baobab Fruit Pulp (*Adansonia digitata*). Food Res. Int..

[22] Irondi E.A., Akintunde J.K., Agboola S.O., Boligon A.A., Athayde M.L. (2017). Blanching Influences the Phenolics Composition, Antioxidant Activity, and Inhibitory Effect of Adansonia Digitata Leaves Extract on Alpha-Amylase, Alpha-Glucosidase, and Aldose Reductase. Food Sci. Nutr..

[23] Tsetegho Sokeng A.J., Sobolev A.P., Di Lorenzo A., Xiao J., Mannina L., Capitani D., Daglia M. (2019). Metabolite Characterization of Powdered Fruits and Leaves From *Adansonia digitata* L. (Baobab): A Multi-Methodological Approach. Food Chem..

[24] Ismail B.B., Pu Y., Guo M., Ma X., Liu D. (2019). LC-MS/QTOF Identification of Phytochemicals and the Effects of Solvents on Phenolic Constituents and Antioxidant Activity of Baobab (*Adansonia dgitata*) Fruit Pulp. Food Chem..

[25] Ismail B.B., Pu Y., Fan L., Dandago M.A., Guo M., Liu D. (2019). Characterizing the Phenolic Constituents of Baobab (*Adansonia digitata*) Fruit Shell By LC-MS/QTOF and Their In Vitro Biological Activities. Sci. Total. Environ..

[26] Cicolari S., Dacrema M., Tsetegho Sokeng A.J., Xiao J., Atchan Nwakiban A.P., Di Giovanni C., Santarcangelo C., Magni P., Daglia M. (2020). Hydromethanolic Extracts from Adansonia digitata L. Edible Parts Positively Modulate Pathophysiological Mechanisms Related to the Metabolic Syndrome. Molecules.

[27] Coe S.A., Clegg M., Armengol M., Ryan L. (2013). The Polyphenol-Rich Baobab Fruit (*Adansonia digitata* L.) Reduces Starch Digestion and Glycemic Response in Humans. Nutr. Res..

[28] Suliman H.M., Osman B., Abdoon I.H., Saad A.M., Khalid H. (2020). Ameliorative Activity of Adansonia Digitata Fruit on High Sugar/High Fat Diet-Simulated Metabolic Syndrome Model in Male Wistar Rats. Biomed. Pharmacother..

[29] Ntchapda F., Bonabe C., Atsamo A.D., Kemeta Azambou D.R., Bekono Fouda Y., Imar Djibrine S., Seke Etet P.F., Theophile D. (2020). Effect of Aqueous Extract of Adansonia digitata Stem Bark on the Development of Hypertension in L-NAME-Induced Hypertensive Rat Model. Evid. Based Complement. Alternat. Med..

[30] Owoyele B.V., Bakare A.O. (2018). Analgesic Properties of Aqueous Bark Extract of *Adansonia digitata* in Wistar Rats. Biomed. Pharmacother..

[31] Adeoye A.O., Olanlokun J.O., Tijani H., Lawal S.O., Babarinde C.O., Akinwole M.T., Bewaji C.O. (2019). Molecular Docking Analysis of Apigenin and Quercetin From Ethylacetate Fraction of *Adansonia digitata* With Malaria-Associated Calcium Transport Protein: An In Silico Approach. Heliyon.

[32] Garvey R., Clegg M., Coe S. (2017). The Acute Effects of Baobab Fruit (*Adansonia digitata*) on Satiety in Healthy Adults. Nutr. Health.

[33] Hanafy A., Aldawsari H.M., Badr J.M., Ibrahim A.K., Abdel-Hady Sel S. (2016). Evaluation of Hepatoprotective Activity of Adansonia digitata Extract on Acetaminophen-Induced Hepatotoxicity in Rats. Evid. Based Complement. Alternat. Med..

[34] Ghoneim M.A., Hassan A.I., Mahmoud M.G., Asker M.S. (2016). Protective Effect of Adansonia digitata against Isoproterenol-Induced Myocardial Injury in Rats. Anim. Biotechnol..

[35] Ismail B.B., Yusuf H.L., Pu Y., Zhao H., Guo M., Liu D. (2020). Ultrasound-Assisted Adsorption/Desorption for the Enrichment and Purification of Flavonoids from Baobab (*Adansonia digitata*) Fruit Pulp. Ultrason. Sonochem..

[36] Debelo H., Ndiaye C., Kruger J., Hamaker B.R., Ferruzzi M.G. (2020). African *Adansonia digitata* Fruit Pulp (Baobab) Modifies Provitamin A Carotenoid Bioaccessibility from Composite Pearl Millet Porridges. J. Food Sci. Technol..

[37] Braca A., Sinisgalli C., De Leo M., Muscatello B., Cioni P.L., Milella L., Ostuni A., Giani S., Sanogo R. (2018). Phytochemical Profile, Antioxidant and Antidiabetic Activities of *Adansonia digitata* L. (Baobab) from Mali, as a Source of Health-Promoting Compounds. Molecules.

[38] Al-Juhaimi F., Babtain I.A., Mohamed Ahmed I.A., Alsawmahi O.N., Ghafoor K., Adiamo O.Q., Babiker E.E. (2020). Assessment of Oxidative Stability and Physicochemical, Microbiological, and Sensory Properties of Beef Patties Formulated With Baobab Seed (*Adansonia Digitata*) Extract. Meat Sci..

[39] Obizoba I.C., Anyika J.U. (1994). Nutritive Value of Baobab Milk (Gubdi) and Mixtures of Baobab (*Adansonia digitata* L.) and Hungry Rice, Acha (*Digitaria exilis*) Flours. Plant Foods Hum. Nutr..

[40] A.O.A.C (2000). Official Methods of Analysis of the Aoac.

[41] FDA (2005). Food and Drug Administration (2002).

[42] (2021). Microbiology of the Food Chain—Horizontal Method for the Enumeration of Coagulase-Positive Staphylococci (Staphylococcus aureus and Other Species)—Part 1: Method Using Baird-Parker Agar Medium.

[43] Bettaieb I., Bourgou S., Wannes W.A., Hamrouni I., Limam F., Marzouk B. (2010). Essential Oils, Phenolics, and Antioxidant Activities of Different Parts of Cumin (*Cuminum cyminum* L.). J. Agric. Food Chem..

[44] Hassan M.F., Barakat H. (2018). Effect of Carrot and Pumpkin Pulps Adding on Chemical, Rheological, Nutritional and Organoleptic Properties of Ice Cream. Food Nutr. Sci..

[45] Mohdaly A.A.A., Hassanien M.F.R., Mahmoud A., Sarhan M.A., Smetanska I. (2012). Phenolics Extracted from Potato, Sugar Beet, and Sesame Processing By-Products. Int. J. Food Prop..

[46] Kumaran A., Karunakaran R.J. (2007). In vitro Antioxidant Activities of Methanol Extracts of Five Phyllanthus Species from India. LWT—Food Sci. Technol..

[47] Muse M., Hartel R.W. (2004). Ice Cream Structural Elements that Affect Melting Rate and Hardness. J. Dairy Sci..

[48] Lavelli V., Corey M., Kerr W., Vantaggi C. (2011). Stability and Anti-Glycation Properties of Intermediate Moisture Apple Products Fortified With Green Tea. Food Chem..

[49] Gouda M., Sheng L., Aadil R.M., Liu Y., Ma M., Li X., He Y., Munekata P.E., Lorenzo J.M. (2021). Interaction of Bioactive Mono-Terpenes with Egg Yolk on Ice Cream Physicochemical Properties. Foods.

[50] Carroll R.J., Thompson M.P., Nutting G.C. (1968). Glutaraldehyde Fixation of Casein Micelles for Electron Microscopy. J. Dairy Sci..

[51] Steel R.G. (1997). Pinciples and Procedures of Statistics a Biometrical Approach.

[52] Burak Çınar Ş., Çalışkan Koç G., Dirim S.N., Ünal G., Akalın A.S. (2020). Textural and Sensorial Characteristics of Set-Type Yogurt Containing *Bifidobacterium animalis* Subsp. Lactis Bb-12 and Quince Powder. J. Food Meas. Charact..

[53] Umelo M., Uzoukwu A., Odimegwu E., Agunwah I., Njoku N., Alagbaoso S. (2014). Proximate, Physicochemical and Sensory Evaluation of Ice Cream From Blends of Cow Milk and Tigernut (*Cyperus esculentus*) Milk. Int. J. Sci. Res. Innov. Technol..

[54] Abdullahi M., Zainab F.A., Pedavoah M., Bashir U., Sumayya U., Ibrahim A. (2014). Evaluating the Suitability of *Adansonia digitata* Fruit Pulp for the Production of Yoghurt. Int. J. Biol. Chem. Sci..

[55] Murray S.S., Schoeninger M.J., Bunn H.T., Pickering T.R., Marlett J.A. (2001). Nutritional Composition of Some Wild Plant Foods and Honey Used by Hadza Foragers of Tanzania. J. Food Compos. Anal..

[56] Ahmed A.A.-H., Sayed R.G., Sayed M. (2014). Nutritional Value and Sanitary Evaluation of Raw Camel’s Milk. Emir. J. Food Agric..

[57] Dabora S.A.M.A. (2016). Assessment of the Effect of Addition of Baobab (Adansonia digitata L.) Fruit Pulp on Properties of Camel Milk Yoghurt.

[58] Goff H.D. (2006). Ice Cream. Advanced Dairy Chemistry Volume 2 Lipids.

[59] Nalbone L., Vallone L., Giarratana F., Virgone G., Lamberta F., Marotta S.M., Donato G., Giuffrida A., Ziino G. (2022). Microbial Risk Assessment of Industrial Ice Cream Marketed in Italy. Appl. Sci..

[60] Ciosek Ż., Kot K., Kosik-Bogacka D., Łanocha-Arendarczyk N., Rotter I. (2021). The Effects of Calcium, Magnesium, Phosphorus, Fluoride, and Lead on Bone Tissue. Biomolecules.

[61] Kogure M., Nakaya N., Hirata T., Tsuchiya N., Nakamura T., Narita A., Suto Y., Honma Y., Sasaki H., Miyagawa K. (2021). Sodium/Potassium Ratio Change Was Associated With Blood Pressure Change: Possibility of Population Approach for Sodium/Potassium Ratio Reduction in Health Checkup. Hypertens. Res..

[62] Kamanula M. (2018). Mineral and Phytochemical Composition of Baobab (*Adansonia digitata* L.) Root Tubers from Selected Natural Populations of Malawi. Malawi Med. J..

[63] Barakat H. (2021). Nutritional and Rheological Characteristics of Composite Flour Substituted With Baobab (*Adansonia Digitata* L.) Pulp Flour for Cake Manufacturing and Organoleptic Properties of Their Prepared Cakes. Foods.

[64] Arellano F.E., Braeuer S., Fernández Cirelli A., Goessler W., Pérez Carrera A.L. (2019). Occurrence of Major and Trace Elements in Powdered Milk From Argentina. Int. J. Dairy Technol..

[65] Sun-Waterhouse D., Edmonds L., Wadhwa S.S., Wibisono R. (2013). Producing Ice Cream Using A Substantial Amount of Juice From Kiwifruit with Green, Gold or Red Flesh. Food Res. Int..

[66] da Silva Dias J.C. (2014). Nutritional and Health Benefits of Carrots and their Seed Extracts. Food Nutr. Sci..

[67] Şimşek B., İlhan G. (2021). Some Physicochemical, Rheological and Sensory Properties of Flavored Ice Cream. Niğde Ömer Halisdemir Üniversitesi Mühendislik Bilim. Derg..

[68] Milovanovic B., Djekic I., Miocinovic J., Djordjevic V., Lorenzo J.M., Barba F.J., Mörlein D., Tomasevic I. (2020). What Is the Color of Milk and Dairy Products and How Is It Measured?. Foods.

[69] Odoom D. (2021). Characterization of Baobab (*Adansonia digitata* L.) in the Builsa District of Ghana. J. Food Technol. Preserv..

[70] Barros E.L.D.S., Silva C.C., Canella M.H.M., Verruck S., Prestes A.A., Vargas M.O., Maran B.M., Esmerino E.A., Silva R., Balthazar C.F. (2021). Effect of Replacement of Milk by Block Freeze Concentrated Whey in Physicochemical and Rheological Properties of Ice Cream. Food Sci. Technol..

[71] Amador J., Hartel R., Rankin S. (2017). The Effects of Fat Structures and Ice Cream Mix Viscosity on Physical and Sensory Properties of Ice Cream. J. Food Sci..

[72] Cavender G.A., Kerr W.L. (2020). Microfluidization of Full-Fat Ice Cream Mixes: Effects on Rheology and Microstructure. J. Food Eng..

[73] Mortazavian A.M., Kheynoor N., Pilevar Z., Sheidaei Z., Beikzadeh S., Javanmardi F. (2020). Rheological Characteristics and Methodology of Ice Cream: A Review. Curr. Nutr. Food Sci..

[74] Hasan G.M., Saadi A.M., Jassim M.A. (2020). Study the Effect of Replacing the Skim Milk Used in Making Ice Cream with Some Dried Fruit. Food Sci. Technol..

[75] Karlsson A.O., Ipsen R., Ardö Y. (2007). Observations of Casein Micelles in Skim Milk Concentrate by Transmission Electron Microscopy. LWT—Food Sci. Technol..

[76] McKenna A.B., Lloyd R.J., Munro P.A., Singh H. (1999). Microstructure of Whole Milk Powder and of Insolubles Detected by Powder Functional Testing. Scanning.

[77] Hassan J.A., El Zubeir I.E.M. (2020). Effect Of Vanilla, Baobab (*Adanosonia digitata*) and Papaya (*Carica Papaya*) Fruits on the Microbiological and Sensory Propertis of Camel Milk Ice Cream. Annals. Food Sci. Technol..

